# Non-Anemic Iron Deficiency Predicts COPD Exacerbations and Hospitalizations: Results from a Prospective Cohort

**DOI:** 10.3390/jcm14124154

**Published:** 2025-06-11

**Authors:** Carlos A. Amado, Cristina Ghadban, Juan Agüero, Bernardo A. Lavín, Paula Martín-Audera, Armando R. Guerra, Ana Berja, Nieves Aranda, Anastasia Guzun, Ana Isabel Insua, Mayte García-Unzueta

**Affiliations:** 1 Department of Pulmonology, Hospital Universitario Marqués de Valdecilla, 39005 Santander, Spainnieves.aranda@idival.org (N.A.); 2 Department of Medicine and Psychiatry, University of Cantabria, 39005 Santander, Spain; 3 IDIVAL (Instituto de Investigación Biomédica de Cantabria), 39011 Santander, Spainanainsual.1994@gmail.com (A.I.I.); 4 Department of Biochemistry, Hospital Universitario Marqués de Valdecilla, 39005 Santander, Spain; 5 Department of Biochemistry, Hospital Universitario de Burgos, 09006 Burgos, Spain; pmartinaudera@gmail.com

**Keywords:** COPD, exacerbation, NAID, exercise capacity, iron

## Abstract

**Background:** Non-anemic iron deficiency (NAID) has been increasingly recognized as a potential factor affecting chronic obstructive pulmonary disease (COPD) outcomes. However, its prognostic role in COPD exacerbations and hospitalizations remains poorly understood. This study aimed to evaluate the prevalence of NAID in COPD patients, its impact on functional parameters, and its predictive value for exacerbations and hospitalizations. **Methods:** This prospective observational study included 238 patients with stable COPD and 60 age- and sex-matched smokers without COPD as a control group. NAID was defined as serum ferritin < 100 ng/mL or serum ferritin between 100 and 299 ng/mL with transferrin saturation < 20%. Clinical assessments included pulmonary function tests, 6 min walk distance (6MWD), handgrip strength, and fat-free mass index (FFMI). Patients were followed for 12 months to record moderate and severe COPD exacerbations. Cox regression analysis was used to determine the predictive value of NAID for exacerbations and hospitalizations. **Results:** NAID was present in 68.9% of COPD patients compared to 46.7% of smokers without COPD (*p* = 0.001). COPD patients with NAID had lower 6MWD (430 (330–500) m vs. 462 (390–510) m, *p* = 0.029), reduced FFMI (17.9 (15.5–20.2) kg/m^2^ vs. 20.6 (17.6–22.6) kg/m^2^, *p* < 0.001), and weaker handgrip strength (26 (22–33) kg vs. 34 (27–40) kg, *p* < 0.001) compared to non-NAID COPD patients. During the 12-month follow-up period, 140 patients developed moderate COPD exacerbations (107 in the NAID group), and 43 patients were hospitalized due to severe exacerbations (36 in the NAID group). Cox regression analysis showed that NAID was an independent predictor of moderate COPD exacerbations (HR 1.846, 95% CI 1.249–2.729, *p* = 0.002) and hospitalization (HR 2.537, 95% CI 1.129–5.703, *p* = 0.024) after adjusting for age, sex, lung function, and comorbidities. **Conclusions:** NAID is highly prevalent in COPD and is associated with worse exercise capacity, lower muscle mass, and increased exacerbation risk independently of sex and age. These findings suggest that NAID could be a valuable biomarker for risk stratification in COPD patients, warranting further research on potential therapeutic interventions targeting iron metabolism.

## 1. Introduction

Chronic obstructive pulmonary disease (COPD) is a leading cause of morbidity and mortality worldwide, characterized by persistent airflow limitation [1]. COPD dysregulates systemic inflammation [2], oxidative stress [3,4], and diverse metabolic pathways [5,6], generating systemic manifestations that contribute to disease progression and worse clinical outcomes. Iron homeostasis may also play a critical role in disease pathophysiology [7], especially given its interplay with erythropoietin regulation [8] and oxygen transport in chronic hypoxemia.

Iron is an essential micronutrient involved in oxygen transport, mitochondrial function, and cellular metabolism. Non-anemic iron deficiency (NAID), defined as low iron stores without concomitant anemia [9], has emerged as a clinically relevant condition in chronic diseases such as heart failure [10]. In COPD, NAID has been linked to lower physical activity [11], spirometric variables [12,13], exercise capacity [14], a diminished response to pulmonary rehabilitation [14], or previous exacerbations [15].

However, most available data come from cross-sectional analyses, limiting the ability to determine whether NAID contributes to COPD progression or is merely a consequence of systemic inflammation. Longitudinal studies assessing the prognostic significance of NAID in COPD exacerbations and hospitalizations are lacking.

This prospective cohort study aims to evaluate the prevalence of NAID in a well-characterized COPD population and determine its impact on functional capacity, exacerbations, and hospitalizations over a 12-month follow-up period. We hypothesize that NAID is an independent predictor of moderate and severe COPD exacerbations in COPD.

## 2. Methods

### 2.1. Study Design

This prospective observational study was conducted at a tertiary hospital in Spain from October 2018 to September 2023. The samples and patient data utilized in this study were stored at the Biobank Valdecilla (PT17/0015/0019). The COPD collection received approval from our institution’s Ethics Committee (2018.189). The study protocol has been registered on ClinicalTrials.gov (https://clinicaltrials.gov/study/NCT06102993 (accessed on 10 January 2025)) and was also reviewed and approved by our institution’s Ethics Committee (approval number 2023.297). Prior to participation, all individuals provided written informed consent.

### 2.2. Participants

Patients with a COPD diagnosis were randomly selected during their regular appointments at the specialized outpatient clinic. Control participants, matched by age and sex, consisted of smokers without a COPD diagnosis and were recruited from our institution’s smoking cessation clinics. COPD was diagnosed according to the Global Initiative for Chronic Obstructive Lung Disease (GOLD) criteria [1], defined as a post-bronchodilator FEV_1_/FVC ratio < 0.7. NAID was defined as serum ferritin < 100 ng/mL or serum ferritin between 100 and 299 ng/mL with transferrin saturation < 20% [7,11,14].

Inclusion criteria were diagnosis of COPD according to GOLD [1] or being an age- and sex-matched smoker without COPD.

Exclusion criteria were (1) COPD exacerbation within eight weeks prior to study inclusion; (2) participation in pulmonary rehabilitation within six months before enrollment; (3) previous diagnosis of coronary artery disease, active cancer, or severe renal dysfunction (glomerular filtration rate < 50 mL/min/1.73 m^2^); (4) presence of polycythemia (Hb > 16 g/dL in women or >16.5 g/dL in men) [15], myeloproliferative neoplasms [16] anemia (Hb < 12 g/dL in women or <13 g/dL in men) [17], or genetic disorders affecting iron metabolism (e.g., hemochromatosis); or (5) patients in treatment with iron supplementation.

### 2.3. Clinical and Functional Assessment

Spirometry and diffusing capacity for carbon monoxide (DLCO) were performed following Spanish Society of Pulmonology and Thoracic Surgery (SEPAR) guidelines [18], measuring FEV_1_, FVC, and FEV_1_/FVC pre- and post-bronchodilator. Exercise capacity was assessed using the six-minute walk distance (6MWD) test [19]. Handgrip strength was measured using a GRIP-A hand dynamometer (Takei, Niigata, Japan).

Anthropometric data included body mass index (BMI) and fat-free mass index (FFMI), measured via bioelectrical impedance OMRON BF511 (Omron, Kyoto, Japan). Dyspnea severity was determined using the modified Medical Research Council (mMRC) scale, and symptom burden was assessed with the COPD Assessment Test (CAT).

### 2.4. Laboratory Analysis

Venous blood samples were collected in the morning after an overnight fast. Iron metabolism markers (serum iron, ferritin, transferrin, and transferrin saturation) were analyzed using Siemens traceable enzymatic method assays Atellica Analyzer (Siemens, Munich. Germany). Hemoglobin and hematocrit were determined using a DxH 900 analyzer (Beckman Coulter. Inc., Brea, CA, USA). Transferrin receptor (sTfR1) was measured using the Human sTfR1 ELISA Kit EH0386 (FineTest, Wuhan, China) as per the manufacturer’s instructions.

### 2.5. Follow-Up and Outcomes

Participants were prospectively followed for 12 months to assess moderate and severe COPD exacerbations. Exacerbations were recorded during scheduled 6- and 12-month visits and confirmed through medical records. Exacerbations were considered moderate if they required antibiotics and/or systemic corticosteroids. Severe exacerbation was defined as exacerbations leading to hospitalization. These definitions are based on GOLD criteria.

### 2.6. Statistical Analysis

Data were analyzed using SPSS (version 25.0, IBM, NY, USA). Continuous variables were expressed as mean ± standard deviation or median (interquartile range), and categorical variables as percentages. Prior to data collection, we conducted a power analysis using G*Power version 3.0 to estimate the required sample size to detect differences in NAID prevalence between groups. Based on previous studies [7,11,12,13,14,15], assuming a prevalence of 65% in COPD and 45% in controls, a sample of 200 COPD patients and 50 controls was sufficient to achieve 80% power at a 5% significance level. Our final sample (238 COPD patients and 60 controls) exceeded this threshold. Intergroup comparisons were performed using unpaired *t*-tests for parametric data and Mann-Whitney for nonparametric data. Correlations were performed using Pearson’s or Spearman’s correlation coefficients, depending on variable distribution. Logistic regression was used to assess associations between NAID and clinical characteristics, adjusting for confounders. Cox regression was applied to identify independent predictors of exacerbations and hospitalizations, adjusting for relevant clinical variables. A *p*-value < 0.05 was considered statistically significant.

## 3. Results

### 3.1. Baseline Characteristics

**Figure 1:**
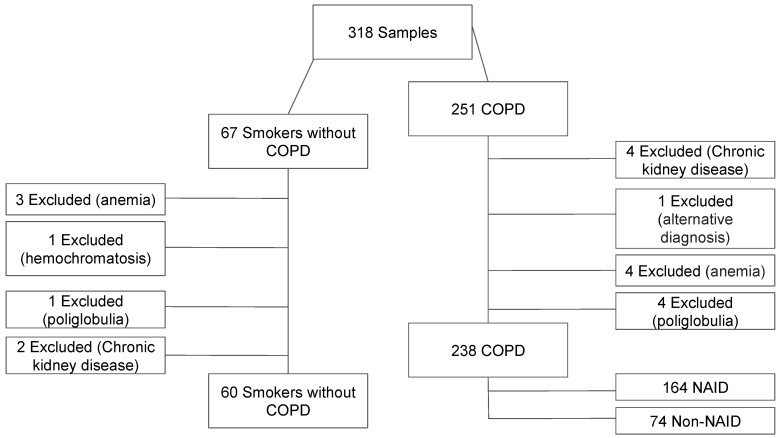
Flowchart for patient selection.

A total of 238 COPD patients and 60 age- and sex-matched smokers without COPD were included in the study. Among the COPD cohort, 164 patients (68.9%) were classified as having non-anemic iron deficiency (NAID) (Figure 1). The demographic and clinical characteristics of the study population are summarized in Table 1.

As expected, COPD patients presented with significantly lower FVC (2938 ± 862 mL vs. 3403 ± 917 mL, *p* = 0.001), FEV_1_ (1390 (985–1900) mL vs. 2595 (2072–3150) mL, *p* < 0.001), and exercise capacity with a 6 min walk distance (6MWD) (447 (348–500) meters, vs. 522 (423–570) meters, *p* < 0.001) compared to controls. COPD patients also exhibited lower fat-free mass index (FFMI) (18.6 (16.0–20.4) kg/m^2^ vs. 19.0 (16.4–20.8) kg/m^2^, *p* < 0.001) and a higher symptom burden, as reflected by the higher COPD Assessment Test (CAT) scores (10 (6–16) vs. 3 (1–6), *p* < 0.001). Ninety-one (38%) COPD patients were treated with triple therapy (inhaled corticosteroids [ICS], LAMA, and LABA); the remaining patients received dual bronchodilation. No significant differences were found in the number of patients treated with ICS when comparing COPD patients with and without NAID.

Within the COPD cohort, patients with NAID exhibited significantly lower FEV_1_ (1320 (940–1800) mL vs. 1575 (1122–2110) mL, *p* = 0.006) and lower FFMI (17.9 (15.5–20.2) kg/m^2^ vs. 20.6 (17.6–22.6) kg/m^2^, *p* < 0.001) than those without NAID. 6MWD was also reduced in the NAID group (430 (330–500) m vs. 462 (390–510) m, *p* = 0.029). Notably, the NAID group had a higher proportion of female patients compared to the non-NAID group (54.3% males vs. 85.1% males, *p* < 0.001). Even though diffusing capacity for carbon monoxide (DLCO) was slightly lower in NAID patients, the difference did not reach statistical significance. There was a higher prevalence of GOLD E (exacerbators) in the NAID group.

### 3.2. Iron Metabolism Markers in COPD Patients

As expected, NAID patients had significantly lower serum iron (88.6 ± 35.7 µg/dL vs. 108.1 ± 26.3 µg/dL, *p* < 0.001) and ferritin levels (47.4 (25.1–82.0) ng/mL vs. 216 (163–318) ng/mL, *p* < 0.001) than those without NAID. Transferrin saturation was also reduced in NAID patients (24.2 ± 10.4% vs. 32.2 ± 8.0%, *p* < 0.001), while soluble transferrin receptor (sTfR1) levels did not differ significantly between groups (*p* = 0.221).

### 3.3. Association Between NAID and COPD Severity

Univariate logistic regression analysis (Table 2) showed that NAID was associated with lower FFMI (OR 0.707; 95% CI 0.620–0.806, *p* < 0.001) and lower 6MWD (OR 0.996; 95% CI 0.994–0.999, *p* = 0.005). In the multivariate model, these associations remained significant for FFMI (OR 0.750; 95% CI 0.637–0.884, *p* = 0.001) and 6MWD (OR 0.995; 95% CI 0.991–0.999, *p* = 0.010) (Table 2).

### 3.4. NAID as a Predictor of COPD Exacerbations

During the 12-month follow-up period, 140 patients developed moderate COPD exacerbations (107 in the NAID group) and 43 patients were hospitalized due to severe exacerbations (36 in the NAID group).

**Figure 2:**
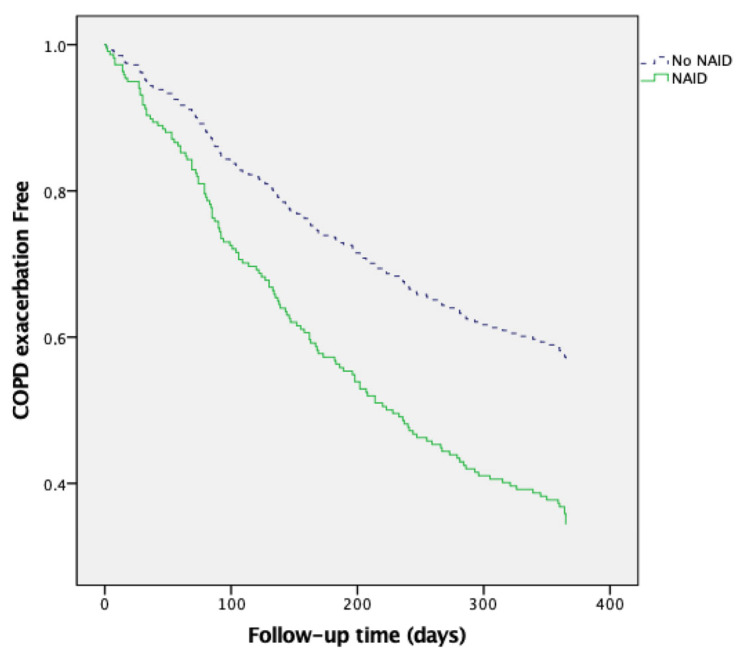
NAID as a predictor of moderate COPD exacerbations.

**Figure 3:**
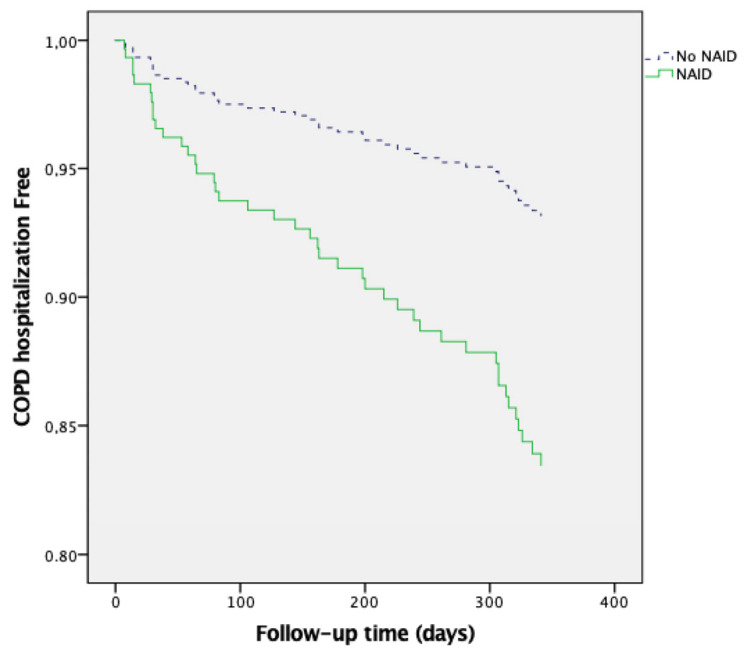
NAID as a predictor of severe COPD exacerbations.

The results of univariate Cox proportional risk analysis revealed that NAID was a predictor of moderate exacerbations (HR 1.846, 95% CI 1.249–2.729, *p* = 0.002) and hospitalization (HR 2.537, 95% CI 1.129–5.703, *p* = 0.024). Multivariate Cox regression analysis (Table 3, Figure 2) revealed that NAID was associated with an increased risk of moderate exacerbations (HR 1.843; 95% CI 1.225–2.772, *p* = 0.003), independent of age, sex, and baseline lung function. Similarly, NAID was significantly associated with a higher risk of hospitalization (HR 2.559; 95% CI 1.105–5.926, *p* = 0.028), independent of age, sex, and baseline lung function (Table 4, Figure 3).

## 4. Discussion

Our study is the first prospective investigation to examine the association between NAID and the subsequent risk of COPD exacerbations. Our findings reveal several key insights. First, patients with stable COPD exhibited a higher prevalence of NAID compared to smokers without COPD. Additionally, NAID in COPD was associated with critical clinical outcomes, including reduced exercise capacity, as measured by 6MWD, and altered body composition, as indicated by FFMI. Moreover, the prospective component of our study demonstrated that COPD patients with NAID face a higher risk of developing moderate to severe exacerbations. Finally, our results suggest that NAID may represent an important treatable trait influencing COPD prognosis.

This is the first study to describe a higher prevalence of NAID in patients with COPD as compared with a group of healthy smokers. This is an interesting finding that can be explained based on several factors. First, this could be attributed to a lower iron intake. This has been described before in a large cross-sectional study that showed that most patients with COPD presented insufficient iron intake [20]. Furthermore, low-grade chronic inflammation (inflammaging), which is a key pathogenic factor in COPD, has been suggested as a potential factor associated with NAID [21,22]. Although patients with severe chronic diseases were excluded from this study, COPD is commonly associated with multiple comorbidities and undiagnosed or mild chronic conditions (e.g., early-stage renal disease, subclinical heart failure, occult malignancies, or gastrointestinal disorders), which may contribute to increased iron loss, impaired absorption, or inflammation-related iron dysregulation.

Our study also showed interesting associations between NAID and clinical characteristics. Women with COPD had a higher prevalence of NAID compared with men; this association was also revealed in the univariate logistic regression analysis but was not confirmed in the multivariate analysis. This interesting finding has not been reported before in the onset of COPD but is concordant with studies performed in older adults from the general population [23,24]. NAID was also associated with lower exercise capacity using 6MWD. These results are consistent with a previous cross-sectional study performed in COPD [15] that did not use multivariate analysis and did not evaluate strength or body composition. Our results are also concordant with findings in other chronic conditions such as chronic heart failure [25] or chronic kidney disease [26]. An association between physical activity and NAID has also been reported [11]; however, physical activity was not assessed in our study. Our study is the first to find an association between lower FFMI and NAID, suggesting that not only exercise capacity is affected by NAID but also muscle mass, which is especially interesting since FFMI is a well-known independent predictor of mortality in COPD [27]. We also showed for the first time that patients with COPD and NAID had lower hand grip strength than COPD patients without NAID.

The prospective nature of the study allowed us to evaluate the impact of NAID in future moderate COPD exacerbations and hospitalizations for the first time. NAID was an important risk factor for both moderate exacerbations and hospitalizations. We believe that this is the most important finding of our study since exacerbations are key clinical outcomes in COPD. This novel finding is concordant with a previous cross-sectional study that found that patients with NAID had more previous exacerbations than patients without NAID [28], also described in our study. Our results are also supported by findings from a large retrospective cohort study [29] that evaluated the relationship between iron deficiency and all-cause hospitalization in patients from a clinical registry. The authors found that low transferrin saturation (TSAT ≤ 20%) was associated with an increased risk of all-cause hospitalization in women, independently of hemoglobin levels, suggesting a clinically relevant role for NAID in COPD prognosis. Although their study did not distinguish hospitalizations due to COPD exacerbations specifically, their results align with our prospective data, reinforcing the idea that NAID may contribute to disease instability and higher healthcare utilization. Importantly, our study expands on these findings by demonstrating that NAID is associated not only with hospitalizations but also with moderate exacerbations, highlighting its potential role as a modifiable risk factor in the clinical management of COPD. The role of iron in COPD could be explained by several pathophysiological mechanisms. Iron plays a central role in cellular energy metabolism, immune function, and oxidative stress regulation [24]. Deficiency, even in the absence of anemia, can impair mitochondrial function and reduce skeletal and respiratory muscle performance, potentially increasing susceptibility to respiratory infections and triggering exacerbations. Furthermore, iron deficiency may dysregulate innate immunity and promote a pro-inflammatory state [30], which are both recognized contributors to exacerbation risk in COPD. Future studies incorporating other iron-related molecules could help clarify the pathophysiological insights of our findings.

Our study has several limitations. As an exploratory single-center study, our findings require validation in other settings and in larger multicenter studies that include participants with diverse sociodemographic backgrounds and comorbidities known to influence iron metabolism. Eventually, clinical trials could be conducted to evaluate the role of iron supplementation in COPD management. Although we applied strict exclusion criteria to remove patients with altered pulmonary function or comorbid conditions unrelated to COPD that could influence iron metabolism (e.g., acute exacerbations, sepsis, severe inflammation, renal insufficiency malabsorption, and iron-related disorders), our findings may not be fully generalizable to all COPD patients. Moreover, we included COPD patients with low-grade inflammation and undiagnosed asymptomatic conditions (e.g., coronary disease, atherosclerosis, and hypertension), though their impact on our results is likely minimal. Also, we did not systematically collect data on the use of antiplatelet agents or use of gastric acid inhibitors, which may contribute to NAID through occult gastrointestinal bleeding. However, no patient showed clinical evidence of active or external bleeding at enrollment. In any case, we consider that the specific cause of iron deficiency (low intake or external losses) does not change the results. Additionally, our study identifies associations rather than establishing causality. Despite these limitations, our findings contribute valuable insights into COPD pathophysiology. Finally, iron status was assessed only at baseline and was not re-evaluated during follow-up. It is possible that changes in iron availability or nutritional status over time could have influenced the relationship between baseline NAID and future exacerbations. However, such changes would most likely attenuate the strength of the observed associations, meaning that the true impact of NAID on clinical outcomes may in fact be greater than reported.

Our study has several strengths. First, it is the only study evaluating NAID using a prospective design. Second, it includes a large, well-characterized cohort of COPD patients representative of our community and a well-characterized sex-, age-, and smoking status-matched control group. Third, in the study, we perform a comprehensive clinical and functional assessment—including CAT, mMRC, FFMI, handgrip strength, and 6MWD—providing a multidimensional view of NAID beyond isolated biochemical parameters. Fourth, while most existing studies are cross-sectional or based on hospital records, our design permitted a 1-year follow-up of moderate exacerbations and hospitalizations, capturing clinically meaningful outcomes. Finally, by excluding patients with anemia and acute exacerbations, we ensured a stable COPD population, enhancing the internal validity of our findings.

## 5. Conclusions

In conclusion, NAID is highly prevalent in COPD and is associated with worse functional capacity and increased exacerbation risk. These findings provide new insights into COPD phenotyping and may help guide personalized treatment approaches targeting iron metabolism. Future trials are needed to determine whether iron supplementation could reduce exacerbations and improve clinical outcomes in NAID patients with COPD.

## Figures and Tables

**Table 1:** Demographic, clinical, and biochemical characteristics of controls, COPD patients, and COPD patients with and without NAID.

Demographic Characteristics and Comorbidities	Control Group *n* = 60	COPD *n* = 238	*p*	NAID and COPD *n* = 164	Non-NAID and COPD *n* = 74	*p*
**Age (years)**	66.1 ± 6.1	66.9 ± 8.1	0.455	66.9 ± 8.2	66.7 ± 7.7	0.796
**Male sex *n* (%)**	37 (61.7)	152 (63.9)	0.752	**89 (54.3)**	**63 (85.1)**	**<0.001**
**Current smokers *n* (%)**	30 (50)	87 (36.6)	0.057	23 (32.1)	64 (39)	0.239
**CAT score**	**3 (1–6)**	**10 (6–16)**	**<0.001**	**11 (7–17)**	**9 (4–14)**	**0.007**
**Charlson**	**1 (0–2)**	**1 (1–2)**	**0.012**	1 (1–2)	1 (1–2)	0.900
**mMRC score 0/I/II/III/IV *n* (%)**	**47 (78.3)/8 (13.3)/5 (8.3)/0 (0)**	**81 (34.0)/69 (29.0)/61 (25.6)/27 (11.3)**	**<0.001**	48 (29.3)/51 (31.1)/45 (27.4)/20 (12.2)	33 (44.6)/18 (24.3)/16 (21.6)/7 (9.5)	0.149
**GOLD 1/2/3/4 *n* (%)**	-	35 (14.7)/112 (47.1)/69 (29.0)/22 (9.2)	-	24 (14.6)/76 (46.3)/51 (31.1)/13 (7.9)	11 (14.9)/36 (48.6)/18 (24.3)/9 (12.2)	0.603
**GOLD A/B/E *n* (%)**	-	60 (25.2)/72 (30.2)/106 (44.5)	-	**33 (20.1)/54(32.9)/77 (47.0)**	**27 (36.5)/18 (24.3)/29 (39.2)**	**0.025**
**1 or more admissions in the previous year *n* (%)**	-	170 (71.4)	-	117 (71.3)	53 (71.6)	0.965
**ICS treatment *n* (%)**	-	91 (38.2)	-	26 (35.1)	65 (39.6)	0.509
**Long-term oxygen therapy *n* (%)**	-	14 (5.88)		9 (5.49)	5 (6.76)	0.768
**DM *n* (%)**	8 (13.3)	28 (11.8)	0.739	6 (8.1)	22 (13.4)	0.240
**HTN *n* (%)**	**11 (18.3)**	**105 (44.1)**	**<0.001**	39 (52.7)	66 (40.2)	0.073
**Dyslipidemia *n* (%)**	16 (26.7)	95 (39.9)	0.058	29 (39.2)	66 (40.2)	0.878
**Previous cardiovascular** **events *n* (%)**	3 (5)	29 (12.2)	0.108	10 (13.5)	19 (11.6)	0.674
**Pulmonary Function and Physical Performance Parameters**	**Control Group *n* = 60**	**COPD *n* = 238**	***p***	**NAID and COPD *n* = 164**	**Non-NAID and COPD *n* = 74**	***p***
**FVC (mL)**	**3403 ± 917**	**2938 ± 862**	**0.001**	**2821 ± 838**	**3197 ± 865**	**0.002**
**FVC (%)**	**102.6 ± 18.6**	**87.8 ± 19.0**	**<0.001**	88.3 ±18.6	86.9 ± 19.9	0.608
**FEV_1_ (mL)**	**2595 (2072–3150)**	**1390 (985–1900)**	**<0.001**	**1320 (940–1800)**	**1575 (1122–2110)**	**0.006**
**FEV_1_ (%)**	**99.3 ± 20.4**	**57.1 ± 20.4**	**<0.001**	56.9 ±19.9	57.7 ± 20.8	0.759
**FEV_1_/FVC**	**75.3 (80–72.5)**	**50.1 (39.9–60.3)**	**<0.001**	48.9 (40.1–58.9)	54.1 (39.4–61.7)	0.263
**DLCO (%)**	**84 (74.3–92.3)**	**65.8 (52.5–81)**	**0.014**	62 (51–81)	69 (56–83)	0.260
**DLCO (mmol/min/kPa)**	**6.595 (5.03–8.13)**	**4.78 (3.51–6.43)**	**0.008**	4.55 (3.43–6.01)	5.91 (3.86–6.71)	0.065
**KCO (%)**	**94 (74–108)**	**70 (44–92)**	**0.042**	74 (56–93)	88 (69–100)	0.590
**KCO (mmol/min/kPa/L)**	**1.33 ± 0.24**	**1.01 ± 0.55**	**0.001**	0.99 ± 0.6	1.05 ± 0.44	0.053
**Weight (Kg)**	74.0 (63.7–88.0)	73 (63.8–84.5)	0.699	**70.5 (61–81)**	**78.8 (66.8–96.1)**	**<0.001**
**BMI (Kg/m^2^)**	27.0 (23.7–31.0)	26.9 (24.0–31.2)	0.758	**26.7 (23.8–30.2)**	**28.7 (24.1–32.8)**	**0.002**
**6MWD (m)**	**522 (423–570)**	**447 (348–500)**	**<0.001**	**430 (330–500)**	**462 (390–510)**	**0.029**
**Maximum hand grip strength (Kg)**	33.5 (22.8–40)	29 (23–36)	0.144	**26 (22–33)**	**34 (27–40)**	**<0.001**
**FFMI (Kg/m^2^)**	**19.0 (16.4–20.8)**	**18.6 (16.0–20.4)**	**<0.001**	**17.9 (15.5–20.2)**	**20.6 (17.6–22.6)**	**<0.001**
**Laboratory Parameters**	**Control Group *n* = 60**	**COPD *n* = 238**	***p***	**NAID and COPD *n* = 164**	**Non-NAID and COPD *n* = 74**	***p***
**Albumin (g/dL)**	**4.7 (4.5–4.9)**	**4.6 (4.4–4.8)**	**0.03**	4.6 (4.3–4.8)	4.6 (4.5–4.9)	0.184
**Creatinine (mg/dL)**	0.82 (0.68–0.97)	0.79 (0.68–0.91)	0.377	**0.78 (0.67–0.89)**	**0.84 (0.71–0.95)**	**0.042**
**CK (UI/L)**	72.5 (52.0–115.3)	80.5 (54.3–117.8)	0.472	79 (52–113)	92.5 (57.5–128.8)	0.236
**Hb (g/dL)**	14.1 ± 1.3	14.0 ± 1.3	0.321	14.514.8 ± 1.2	14.8 ± 1.2	0.536
**NAID *n*(%)**	**28 (46.7)**	**164 (68.9)**	**0.001**	-	-	-
**Iron (µg/dL)**	66.1 ± 6.1	66.9 ± 8.1	0.188	**88.6 ± 35.7**	**108.1 ± 26.3**	**<0.001**
**Ferritin (ng/mL)**	105 (50–203)	78.4 (33.8–167.3)	0.097	**47.4 (25.1–82.0)**	**216 (163–318)**	**<0.001**
**Transferrin (mg/dL)**	243 (223–267)	253 (228–279)	0.085	**258 (239–297)**	**240 (221–256)**	**<0.001**
**Transferrin saturation (%)**	**29.9 ± 11.4**	**26.6 ± 10.4**	**0.037**	**24.2 ± 10.4**	**32.2 ± 8.0**	**<0.001**
**sTFR1 (mg/L)**	**1.853 (0.984–2.872)**	**2.231 (1.393–3.642)**	**0.019**	2.250 (1.464–3.857)	2.102 (1.332–3.236)	0.221

NAID = non-anemic iron deficit (ferritin level < 100 ng/mL or ferritin level between 100 and 299 ng/mL with a transferrin saturation < 20%), FVC = forced vital capacity, FEV1 = forced expiratory volume in the first second, mMRC = modified medical research council dyspnea score, CAT = COPD Assessment Test, ICS = inhaled corticosteroids, DM = diabetes mellitus, HTN = arterial hypertension, GOLD = Global initiative for Chronic Obstructive Lung Disease, BMI = body mass index, FFMI = fat-free mass index, 6MWD = 6-min walk test distance, CRP = C-reactive protein, sTRFR1 = sTfR1 = soluble transferrin receptor-1Hb = hemoglobin. Bold font indicates statistical significance.

**Table 2:** Unadjusted and adjusted associations between chronic obstructive pulmonary disease characteristics and NAID using univariate and multivariate logistic regression.

		NAID (Unadjusted)		NAID (Adjusted)	
		OR (95% CI)	*p*	OR (95% CI)	*p*
Age (years)		1.005 (0.971–1.039)	0.795	0.986 (0.939–1.035)	0.570
Sex					
	Male	1		1	
	Female	**4.826 (2.372–9.820)**	**<0.001**	2.075 (0.716–6.015)	0.179
Smoking status					
	Former	1		1	
	Current	1.419 (0.792–2.544)	0.240	0.790 (0.332–1.879)	0.594
GOLD					
	A or B	1		1	
	E	1.373 (0.786–2.401)	0.266	0.841 (0.393–1.800)	0.656
mMRC Dyspnea score		1.305 (0.986–1.728)	0.063	1.079 (0.682–1.710)	0.745
Charlson		1.098 (0.822–1.468)	0.526	1.227 (0.831–1.811)	0.304
ICS treatment		0.825 (0.466–1.460)	0.509	1.628 (0.718–3.694)	0.244
FFMI (kg/m^2^)		**0.707.022 (0.620–0.806)**	**<0.001**	**0.734 (0.619–0.871)**	**<0.001**
6MWD (m)		**0.996 (0.994–0.999)**	**0.005**	**0.995 (0.991–0.999)**	**0.010**
FEV1 (%)		0.998 (0.985–1.011)	0.758	1.007 (0.978–1.036)	0.645
FVC (%)		1.004 (0.989–1.018)	0.606	1.016 (0.989–1.044)	0.251

NAID = non-anemic iron deficit (ferritin level < 100 ng/mL or ferritin level between 100 and 299 ng/mL with a transferrin saturation < 20%), exacerbations = need for antibiotic or systemic corticosteroids, 6MWD = 6-min walk test distance, ICS = inhaled corticosteroids, FEV1 = forced expiratory volume in the first second, FVC = forced vital capacity, FFMI = fat-free mass index, bold font indicates statistical significance.

**Table 3:** Cox regression analysis of predictors of COPD exacerbation.

	B	Wald	*p*	HR	95% IC	
					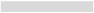 Inferior	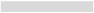 Superior
**Age**	**0.021**	**3.677**	**0.055**	1.022	1.000	1.044
**Sex**	−0.248	1.794	0.180	0.780	0.543	1.122
**Current smoker**	0.068	0.122	0.727	1.071	0.730	1.571
**Charlson**	−0.191	3.706	0.054	0.826	0.680	1.003
**mMRC**	−0.114	1.209	0.271	0.892	0.727	1.094
**FEV1 (% predicted)**	**−0.015**	**8.510**	**0.004**	**0.985**	**0.976**	**0.995**
**GOLD E**	−0.186	1.084	0.298	0.831	0.586	1.178
**NAID**	**0.611**	**8.622**	**0.003**	**1.843**	**1.225**	**2.772**

NAID = non-anemic iron deficit (ferritin level < 100 ng/mL or ferritin level between 100 and 299 ng/mL with a transferrin saturation < 20%), FEV1 = forced expiratory volume in the first second, mMRC = modified Medical Research Council dyspnea score, GOLD E = 2 or more moderate exacerbations or 1 severe exacerbation during previous year. Bold font indicates statistical significance.

**Table 4:** Cox regression analysis of predictors of COPD hospitalization.

	B	Wald	*p*	HR	95% IC	
					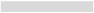 Inferior	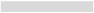 Superior
**Age**	**0.044**	**4.387**	**0.036**	**1.045**	**1.003**	**1.089**
**Sex**	0.048	0.021	0.885	1.050	0.546	2.019
**Current smoker**	<0.001	<0.001	0.999	1.000	0.492	2.029
**Charlson**	−0.019	0.019	0.891	0.981	0.745	1.292
**mMRC**	0.349	3.459	0.063	1.417	0.981	2.047
**FEV1 (% predicted)**	**−0.032**	**8.912**	**0.003**	**0.968**	**0.948**	**0.989**
**GOLD E**	0.323	1.028	0.311	1.381	0.740	2.580
**NAID**	**0.940**	**4.808**	**0.028**	**2.559**	**1.105**	**5.926**

NAID = non-anemic iron deficit (ferritin level < 100 ng/mL or ferritin level between 100 and 299 ng/mL with a transferrin saturation < 20%), FEV1 = forced expiratory volume in the first second, mMRC = modified Medical Research Council dyspnea score, GOLD E = 2 or more moderate exacerbations or 1 severe exacerbation during previous year. Bold font indicates statistical significance.

## Data Availability

Requests to access the datasets should be directed to corresponding author.
